# Optimal CeO_2_ Doping for Synergistically Enhanced Mechanical, Tribological, and Thermal Properties in Zirconia Ceramics

**DOI:** 10.3390/ma19020362

**Published:** 2026-01-16

**Authors:** Feifan Chen, Yongkang Liu, Zhenye Tang, Xianwen Zeng, Yuwei Ye, Hao Chen

**Affiliations:** 1 School of Materials Science and Engineering, Jiangxi University of Science and Technology, Ganzhou 341000, China; 15779635623@163.com (F.C.); 14790989495@163.com (Y.L.); tangzy11229@163.com (Z.T.); 19914724995@163.com (X.Z.); 2 Ganzhou Institute of Tungsten Industrial Technology, Ganzhou 341500, China; 3 Key Laboratory of Efficient Exploitation and Utilization of Tungsten Resources of Jiangxi Province, Ganzhou 341000, China; 4 Engineering Research Center, Ministry of Education, High Efficiency Development and Application of Tungsten Resources, Jiangxi University of Science and Technology, Ganzhou 341000, China

**Keywords:** cerium oxide doping, zirconia, structure, wear, thermal properties

## Abstract

**Highlights:**

**What are the main findings?**

- Optimal performance at 15 wt.% CeO_2_: peak hardness, lowest friction (0.205), and 72.2% lower thermal expansion.
- Enhancements from solid solution strengthening, grain refinement, and phonon scattering via point defects.
- Excess doping (>15 wt.%) leads to CeO_2_ agglomeration, grain coarsening, and increased porosity.

**What are the implications of the main findings?**

- Provides a clear compositional guideline (15 wt.% CeO_2_) for designing high-performance ZrO_2_ ceramics.
- Warns against over-doping, emphasizing precise compositional control for optimal microstructure.
- Enables simultaneous tuning of mechanical strength, wear resistance, and thermal management.

**Abstract:**

CeO_2_ doping is a well-established strategy for enhancing the properties of zirconia (ZrO_2_) ceramics, with the prior literature indicating an optimal doping range of around 10–15 wt.% for specific attributes. Building upon this foundation, this study provides a systematic investigation into the concurrent evolution of mechanical, tribological, and thermophysical properties across a broad compositional spectrum (0–20 wt.% CeO_2_). The primary novelty lies in the holistic correlation of these often separately examined properties, revealing their interdependent trade-offs governed by microstructural development. The 15Ce-ZrO_2_ composition, consistent with the established optimal range, achieved a synergistic balance: hardness increased by 27.6% to 310 HV_1_, the friction coefficient was minimized to 0.205, and the wear rate was reduced to 1.81 × 10^−3^ mm^3^/(N m). Thermally, it exhibited a 72.2% reduction in the thermal expansion coefficient magnitude at 1200 °C and a low thermal conductivity of 0.612 W/(m·K). The enhancement mechanisms are consistent with solid solution strengthening, grain refinement, and likely enhanced phonon scattering, potentially from point defects such as oxygen vacancies commonly associated with aliovalent doping in oxide ceramics, while performance degradation beyond 15 wt.% is linked to CeO_2_ agglomeration and duplex microstructure formation. This work provides a relatively comprehensive insight into the dataset and mechanism, which is conducive to the fine design of multifunctional ZrO_2_ bulk ceramics. It is not limited to determining the optimal doping level, but also aims to clarify the comprehensive performance map, providing reference significance for the development of advanced ceramic materials with synergistically optimized hardness, wear resistance, and thermal properties.

## 1. Introduction

The thrust-to-weight ratio of an aircraft gas turbine increases with the rise in gas temperature within the turbine. This pushes the engine’s operating temperature to the limits of metallic materials. The use of low-thermal-conductivity materials allows the engine to operate at gas temperatures below the melting point of metals, thereby [1] enhancing engine efficiency and performance. To mitigate this, a release layer is necessary. Zirconia (ZrO_2_), characterized by its high hardness, wear resistance, thermal insulation, and corrosion resistance [2], has emerged as an ideal release layer material. Studies have further confirmed its effectiveness in inhibiting spalling and crack formation during sintering [3].

ZrO_2_-based materials find widespread application in fire resistance and high-temperature protection [4,5], owing to their excellent mechanical properties (high strength and toughness) and thermal properties (high melting point, low thermal conductivity), making them a primary choice for high-temperature components. However, challenges include thermal stress arising from poor high-temperature phase stability and thermal expansion coefficient mismatch, as well as diminished thermal insulation performance due to increasing thermal conductivity at elevated temperatures [6]. To address these issues, researchers have pursued modifications such as rare earth doping (e.g., Y_2_O_3_, CeO_2_) [7], composite ceramics (e.g., ZrO_2_-Al_2_O_3_), and nanostructure design, aiming to enhance thermal stability and lower thermal conductivity.

Among these approaches, CeO_2_ doping has been established as an effective modification method, demonstrating three key advantages: (1) significantly enhancing high-temperature phase stability by delaying the tetragonal-to-monoclinic phase transition [7]; (2) optimizing the thermal expansion coefficient to mitigate thermal stress; and (3) suppressing the temperature-dependent rise in thermal conductivity. Compared to conventional Y_2_O_3_-stabilized ZrO_2_, CeO_2_-ZrO_2_ exhibits superior thermal stability, fracture toughness, and oxidation resistance [8]. These properties endow it with significant application potential for graphite boat bulk ceramics and thermal barrier bulk ceramics.

This study investigates the effects of CeO_2_ doping on the physical properties, tribological behavior, and thermal characteristics of zirconia bulk ceramics. An optimal CeO_2_ doping concentration was established, achieving the synergistic enhancement of densification, hardness, wear resistance, and thermal stability. The findings provide a material design strategy for engineering high-performance ZrO_2_-based components that must withstand combined mechanical, frictional, and thermal loads in severe environments, such as in advanced manufacturing, energy conversion, and aerospace systems.

## 2. Experimental Procedure

### 2.1. Materials

The primary materials used in this study were ZrO_2_ powder (AR, 10–20 μm, Shanghai Yaoyi Alloy Materials Co., Ltd., Shanghai, China), CeO_2_ powder (AR, 5 μm, Shanghai Yaoyi Alloy Materials Co., Ltd., Shanghai, China), anhydrous ethanol (purity >99.7%, Xilong Scientific Co., Ltd., Guangzhou, China), and polyacrylic acid (30% solid content, Shanghai Macklin Biochemical Technology Co., Ltd., Shanghai, China).

### 2.2. Sample Preparation

In this experiment, ZrO_2_ was used as the matrix material, and rare earth doped ZrO_2_ ceramics were prepared by adding different contents of CeO_2_ (see Table 1). The specific process is as follows: (1) ZrO_2_ and CeO_2_ powders were weighed according to the ratio of Table 1, ZrO_2_ grinding balls were added at a ball-to-powder ratio of 5:1, and ball milling was performed using anhydrous ethanol as the medium and polyacrylic acid (30 wt.%) as the binder; (2) the composite powder was obtained by vacuum drying, grinding and sieving of ball abrasive; (3) a cylindrical green body was prepared by unidirectional molding with cemented carbide mold (YP-20T hydraulic press, holding pressure for 60 s); and (4) pressureless sintering was carried out in a KSL-1700X box furnace (MTI Corporation, Richmond, CA, USA) under air atmosphere: First, the temperature was raised from 25 °C to 330 °C at a heating rate of 3 °C/min and held for 300 min to remove the polyacrylic acid (PAA) from the sample. Second, the temperature was increased to 1600 °C at a heating rate of 5 °C/min and held for 600 min. Finally, it was cooled to 25 °C at a rate of 5 °C/min in the KSL-1700X.

### 2.3. Characterization

The phase composition of ceramic materials was analyzed by XRD. The X-ray diffraction analysis model used in this experiment was Empyream. The setting parameters of the equipment during the analysis were as follows: the scanning angle was 10–90°, and the scanning speed was 4°/min. Tube voltage: 40 KV, tube current: 40 mA.

The model of the scanning electron microscope used is MLA-650 (Thermo Fisher Scientific, Waltham, MA, USA). It is equipped with energy dispersive spectroscopy (EDS). In order to ensure the clarity of the image, the polished sample is subjected to gold spraying treatment.

In this experiment, the density of rare earth doped ZrO_2_ ceramic materials was determined based on Archimedes’ principle. In this experiment, the same sample was taken and the experiment was repeated three times to obtain the average value. The experiment used the multifunctional electronic hydrometer AU-600ME (Guangdong Hongtuo Instrument Technology Co., Ltd., Dongguan, China) to weigh the mass of the samples in the air and in the water. Calculate the density of the sample according to Formula (1) [9]:

(1) $$D_{1}\;=\;\frac{m_{2}\rho }{m_{1}-m_{2}}$$

In Formula (1), D_1_ is the density of the sample; m_1_ is the mass of the sample in the air, g; m_2_ is the mass of the sample in water, g; and ρ is the density of water at room temperature, g/cm^3^ (usually 1 g/cm^3^).

Using Formula (2), calculate the porosity for each of the three data points obtained from the same sample, and then find the average porosity, and then understand the porosity of ceramic materials [10]:

(2) $$d\;=\;\frac{D_{1}}{D_{2}}\;\times \;100\%$$

In Equation (2), d is the relative density of the sample, %; D_1_ is the density of the sample, g/cm^3^; and D_2_ is the theoretical density of the sample, g/cm^3^.

The hardness value of rare earth doped ZrO_2_ ceramic materials was determined by using the digital Vickers hardness tester THV-50 MDX (Beijing Time Technologies Co., Ltd., Beijing, China), to carry out five hardness tests on the same sample and then take the average value. The parameters were set as pressure load of 1 kg and holding time of 10 s. The value was calculated according to Formula (3) [11]:

(3) $$\mathrm{HV}\;=\;0.891\frac{P}{d_{v}^{2}}$$

In Formula (3), HV is Vickers hardness value, kg/mm^2^; P is the pressure load, kg; and d_v_ is the length of the indentation diagonal, mm.

The HSR-2M(Beijing Jingmeirui Technology Co., Ltd., Beijing, China) reciprocating friction and wear tester was used to determine the friction and wear characteristics of rare earth doped ZrO_2_ ceramic materials. The loading force is 10 N, the frequency is 4 Hz, the total friction time is 8 min, and the one-way round-trip length of friction is 5 mm. After the friction experiment lasted for 8 min, the friction curve stabilized. The maximum and minimum values of the friction coefficient during this stage were taken, and their average was calculated to obtain the average friction coefficient.

At the end of the experiment, the 2D profiles of the wear scars on the surface of the material were measured by a grinding mark tester MT500 (Shandong Yingli Shuo Testing Instrument Co., Ltd., Shangdong, China), and the depth and width of the wear scars were determined. Select different areas on the same wear mark of the sample, and conduct three measurements using the MT500, respectively. Take the average of the three results as the average wear amount of the sample. The data obtained can be used to calculate the wear rate of rare earth doped ZrO_2_ ceramic materials, and the tribological properties of rare earth doped ZrO_2_ ceramic materials are further analyzed and evaluated by the wear rate. In general, the wear rate is calculated according to Formula (4) [12]:

(4) $$K_{V}\;=\;\frac{S\;\times \;l}{N\;\times \;L}$$

K_V_ is the wear rate, mm^3^/(N m); s is the cross-sectional area of the wear scar, mm^2^; l is the length of the wear scar, mm; n is the load force in the friction and wear experiment, N; L is the friction stroke in the friction and wear experiment, m. In this experiment, L = 9.6 m, N = 10 N, and l = 5 mm.

The thermal diffusivity and specific heat capacity of the bulk ceramic were measured by laser thermal conductivity meter (LFA 42, NETZSCH-Gerätebau GmbH, Selb, Germany). The density of the bulk ceramics was measured by the Archimedes drainage method. The test temperature was 1100 °C. The thermal conductivity and thermal diffusivity of three samples with the same content were measured, respectively, and the average values were calculated based on the measurement results. Finally, the thermal conductivity of the bulk ceramic was obtained by Equations (5) and (6) [13,14]:

λ = αC_p_·ρ (5)

C_p_ = 69.2 + 4.3 × 10^−3^T − 1.3 × 10^6^T^−2^ (6)

Here, C_P_ is the specific heat capacity. ρ is the density of the bulk ceramic, which is determined by Archimedes method. α is the thermal diffusion coefficient of the material. λ is the thermal conductivity of the material.

The linear coefficient of thermal expansion (CTE) in the range of 25–1200 °C was measured by a thermal dilatometer (TMA 403 F3, NETZSCH, Bavaria, Germany). The sample was heated from room temperature (T_0_ = 25 °C) to T = 1200 °C at a heating rate of 0.1 °C/s. CTE was calculated by Formula (7) [15]:

(7) $$\alpha \;=\;\Delta L∕\left(L_{0}\;\times \;\Delta L\right)$$

where α is the linear expansion coefficient (unit: K^−1^); ΔL= L − L: length change caused by temperature change (unit: mm); L_0_ is the original length at the initial temperature (the unit is consistent with ΔL); and ΔT = T − T_0_: temperature change (unit: K). Apply Formula (8) to perform relative density-based correction for thermal conductivity:

(8) $$\kappa_{\mathrm{dense}}\;=\;\kappa_{\mathrm{dense}}\;\times \;\frac{\rho_{\mathrm{measured}}}{\rho_{\mathrm{theoretical}}}$$

## 3. Results and Discussion

### 3.1. Phase Composition and Microstructure

**Figure 1:**
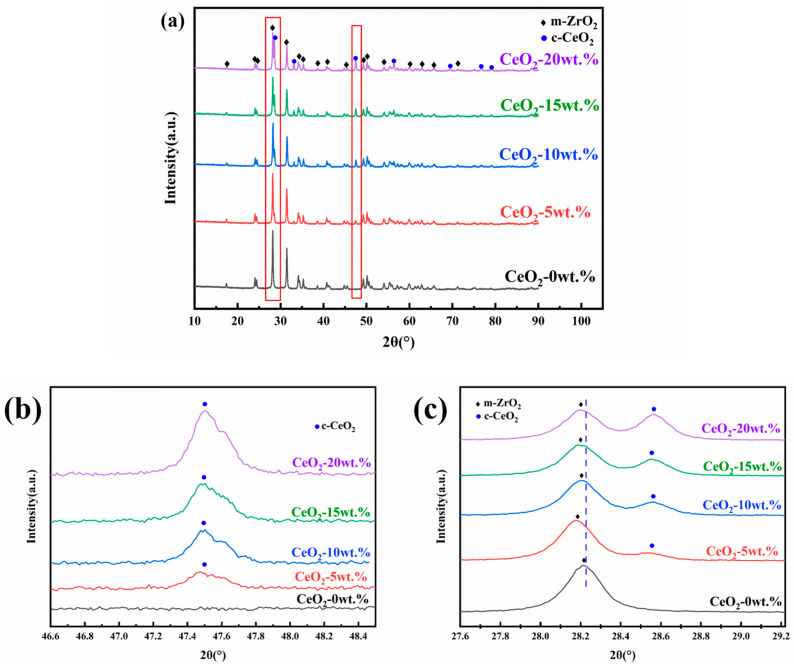
XRD patterns of CeO_2_-doped ZrO_2_ ceramics: (**a**) full range, (**b**,**c**) magnified views.

Figure 1 presents the XRD patterns and corresponding local magnification for CeO_2_ doped ZrO_2_ ceramics with varying CeO_2_ contents after sintering. Comparison with the reference ZrO_2_ pattern revealed the emergence of new diffraction peaks at approximately 28.6°, 33.2°, 47.7°, and 56.2°. The intensity of these new peaks was observed to increase with increasing CeO_2_ content, qualitatively indicating a higher fraction of the secondary CeO_2_ phase. Identification using the relevant PDF card confirmed that these peaks primarily correspond to CeO_2_. While ZrO_2_ exhibits a monoclinic crystal structure at room temperature, CeO_2_ crystallizes in the cubic fluorite structure. When the doping concentration of CeO_2_ exceeds its solid solubility limit in the ZrO_2_ lattice, the excess CeO_2_ precipitates as a distinct secondary phase. This phase separation gives rise to the distinct diffraction peaks observed, which differ from those of the primary ZrO_2_ matrix phase. This finding aligns with the observations reported by Karem et al. [16]. Furthermore, Figure 1c indicates a shift in the characteristic ZrO_2_ peak located near 28.2° towards lower diffraction angles. This shift is attributed to the substitution of Zr^4+^ ions (ionic radius ≈ 0.84 Å for coordination number 8) by the larger Ce^4+^ ions (ionic radius ≈ 0.97 Å for coordination number 8). This ionic substitution leads to an expansion of the unit cell parameters of the ZrO_2_-based solid solution. According to Bragg’s law (nλ = 2 d sin θ, where d is the interplanar spacing, θ is the diffraction angle, λ is the X-ray wavelength, and n is the diffraction order), an increase in d results in a decrease in sin θ (and consequently θ) for a given reflection, manifesting as a peak shift to lower angles [17,18].

**Figure 2:**
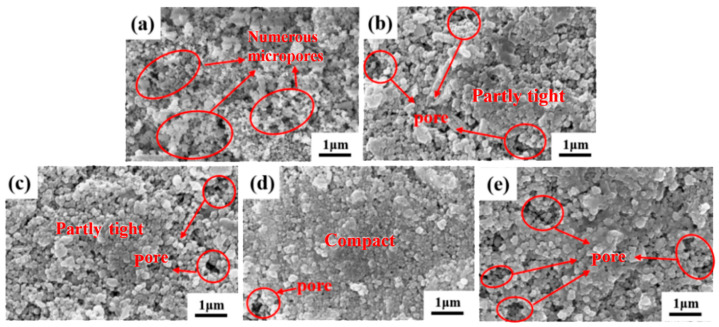
SEM images of CeO_2_-doped ZrO_2_ ceramics: (**a**) 0Ce-ZrO_2_, (**b**) 5Ce-ZrO_2_, (**c**) 10Ce-ZrO_2_, (**d**) 15Ce-ZrO_2_, (**e**) 20Ce-ZrO_2_.

**Figure 3:**
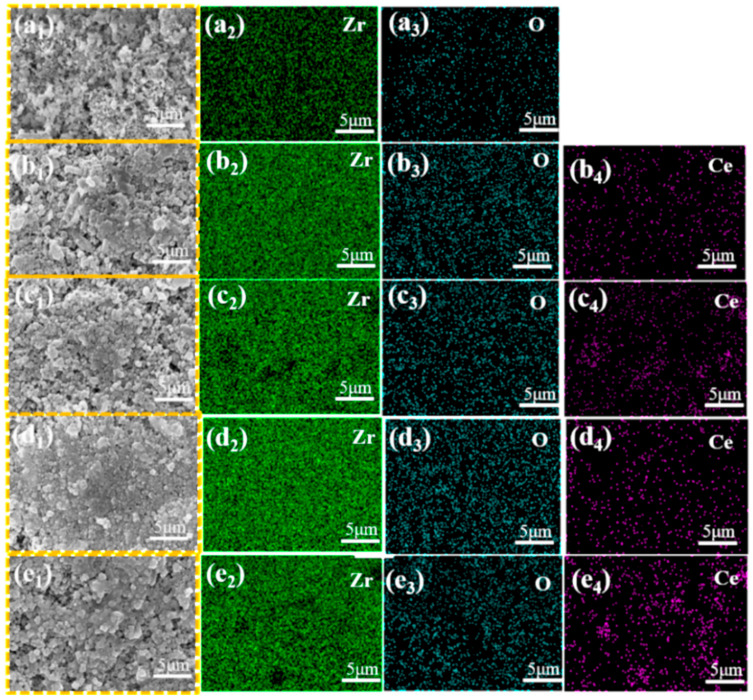
EDS maps of CeO_2_-doped ZrO_2_ ceramics. (**a_1_**–**a_3_**) 0Ce-ZrO_2_ (**b_1_**–**b_4_**) 5Ce- ZrO_2_ (**c_1_**–**c_4_**) 10Ce- ZrO_2_ (**d_1_**–**d_4_**) 15Ce- ZrO_2_ (**e_1_**–**e_4_**) 20Ce-ZrO_2_.

Figure 2 and Figure 3 present the SEM and EDS, respectively, for ZrO_2_ ceramic materials doped with varying CeO_2_ contents. From the SEM images, it can be observed that as the CeO_2_ content increases, the grain size gradually decreases. However, when the CeO_2_ doping level exceeds 15 wt.%, abnormal grain coarsening occurs. This phenomenon can be attributed to two main factors. Firstly, the agglomeration of CeO_2_ nanoparticles reduces the density of effective nucleation sites, promoting grain coalescence and growth [19]. Secondly, the high concentration of CeO_2_ alters sintering behavior, enhancing grain boundary diffusion and thereby facilitating grain coarsening. The residual sintering activity or presence of a local liquid phase contributes to the observed gradual increase in grain size [20]. Correspondingly, the pore number density (or porosity) decreases within the 0–15 wt.% CeO_2_ range, indicating that moderate doping effectively improves microstructural homogeneity and reduces defect density. Conversely, as the CeO_2_ content increases beyond 15 wt.%, the pore number density increases and pores become more widely distributed. This deterioration is attributed to structural inhomogeneities arising from incomplete mass transfer during sintering, particle coalescence issues, and the formation of agglomerates or secondary phases due to the excess dopant, all of which contribute to increased porosity [21]. The EDS elemental mappings in Figure 3 corroborate these findings. The intensity of the Ce^4+^ signal progressively increases with the CeO_2_ doping level. Notably, Figure 3e_4_ reveals localized Ce^4+^ agglomeration/clustering at higher doping concentrations (20 wt.%), directly confirming that excessive CeO_2_ addition promotes particle agglomeration. The O element distribution is uniform across all samples, consistent with the oxide nature of the starting materials. Significantly, the 15Ce-ZrO_2_ sample exhibits the most homogeneous distribution of Zr^4+^, Ce^4+^, and O^2-^ on the material surface, corresponding to its optimal microstructure. At a CeO_2_ doping level of 20 wt.%, both XRD and SEM/EDS analyses indicate the formation of a dual-phase structure in the material. The characteristic diffraction peaks of CeO_2_ in the XRD pattern are significantly enhanced, confirming that it has exceeded the solid solubility limit and precipitated as a secondary phase. SEM/EDS further reveals local agglomeration of Ce elements, forming CeO_2_-rich phase regions, which, together with the ZrO_2_ matrix, constitute a two-phase coexisting system. This structural transformation directly influences the material’s properties and stands in sharp contrast to the uniform single-phase strengthening mechanism achieved through solid solution strengthening and grain refinement in the 15 wt.% sample.

### 3.2. Density, Porosity and Relative Density

**Figure 4:**
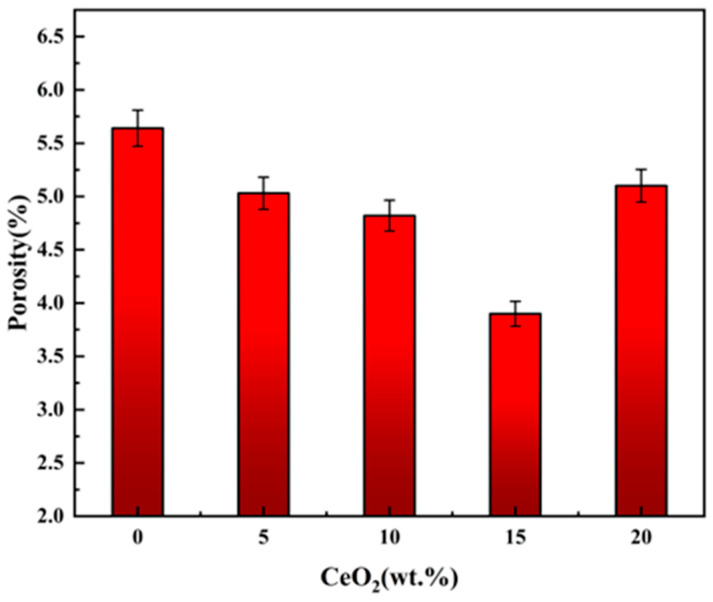
Porosity of CeO_2_-doped ZrO_2_ ceramics.

**Figure 5:**
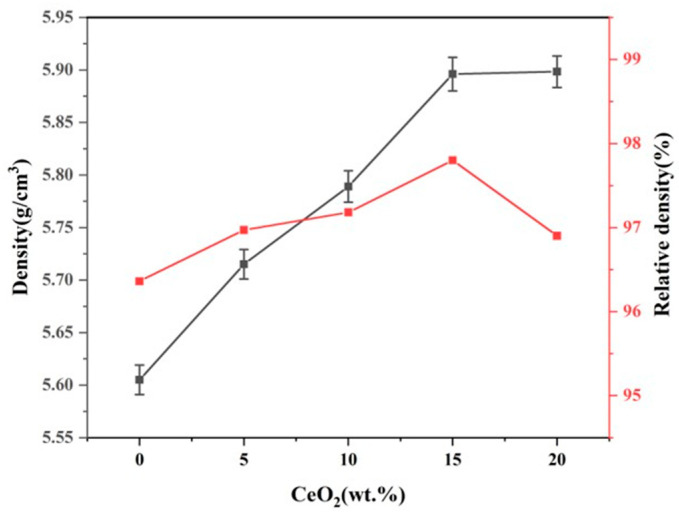
Density and relative density of CeO_2_-doped ZrO_2_ ceramics.

Figure 4 presents the porosity of ZrO_2_ ceramics doped with varying CeO_2_ contents, and Figure 5 presents the bulk density and relative density of ZrO_2_ ceramics doped with varying CeO_2_ contents. Analysis combining Figure 4 (porosity) and Figure 5 reveals that both the pore number density (or porosity) of the Ce-doped ZrO_2_ system exhibit a non-monotonic trend, initially decreasing and subsequently increasing with increasing CeO_2_ content. The reduction in grain size observed with moderate CeO_2_ doping (0–15 wt.%) is primarily attributed to grain boundary pinning effects. CeO_2_ additions interact with the ZrO_2_ matrix grain boundaries, effectively inhibiting grain boundary migration kinetics by altering the grain boundary chemical potential, thereby suppressing grain growth [22]. Furthermore, the uniform distribution of CeO_2_ facilitates microstructural refinement through mechanisms including second-phase dispersion strengthening, grain boundary segregation, and heterogeneous nucleation, leading to reduced porosity and finer grains [23,24,25]. As shown, the bulk density increases with increasing CeO_2_ content. Notably, the rate of density increase diminishes when the CeO_2_ content reaches 15 wt.%. In contrast, the relative density exhibits a non-monotonic trend, initially increasing to a maximum of 96.1% at 15 wt.% CeO_2_, and then decreasing at higher dopant levels. The initial increase in relative density (up to 15 wt.% CeO_2_) can be attributed to two primary factors. (1) Lattice distortion: The significant difference in ionic radius between Ce^4+^ (≈0.97 Å for CN = 8) and Zr^4+^ (≈0.84 Å for CN = 8) induces substantial lattice distortion upon substitution into the ZrO_2_ lattice. This distortion can promote densification mechanisms during sintering [20,26]. (2) Reduced porosity: As discussed in conjunction with Figure 4, the number density of pores decreases within this doping range, directly contributing to the higher relative density. However, when the CeO_2_ content exceeds 15 wt.%, the relative density declines. This deterioration is mainly due to diminished grain boundary pinning and impeded densification. Firstly, excess CeO_2_ can compromise its effectiveness as a grain boundary pinning agent. This reduction in pinning force facilitates abnormal grain growth, leading to the development of larger intergranular pores/voids [27,28]. Secondly, high CeO_2_ concentrations increase the residual oxygen content within the material, which can hinder the final stages of sintering densification. Furthermore, at elevated temperatures, excess CeO_2_ promotes the coalescence of micropores, further reducing the relative density [28].

### 3.3. Hardness, Friction and Wear Performance

**Figure 6:**
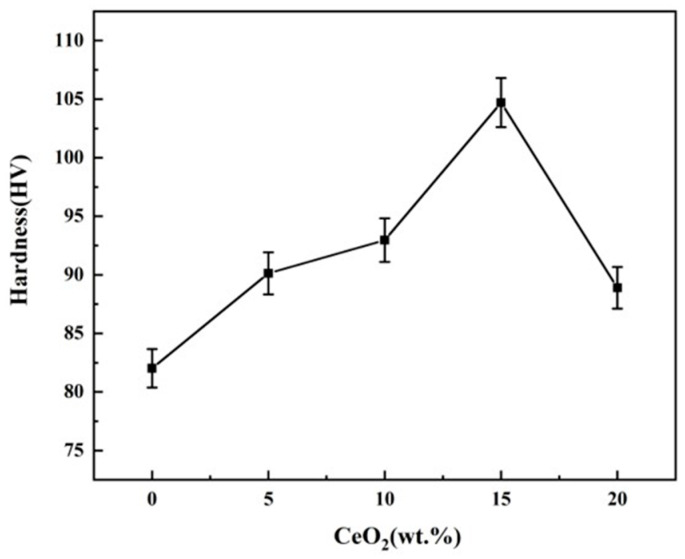
Hardness of CeO_2_-doped ZrO_2_ ceramics.

Figure 6 presents the Vickers hardness (HV_1_) of ZrO_2_ ceramics as a function of CeO_2_ doping content. This HV hardness testing method is feasible, as demonstrated by the tests conducted by Yahui He et al. [2]. The hardness exhibits a non-monotonic dependence on dopant concentration, initially increasing to a maximum value of 310 HV_1_ at 15 wt.% CeO_2_, representing a 27.64% enhancement compared to undoped ZrO_2_, followed by a decrease at higher doping levels. It should be noted that the absolute hardness values obtained in this study (e.g., ~310 HV_1_ for the optimal 15Ce-ZrO_2_) are below the typical range reported for fully dense, fine-grained YSZ ceramics (>1000 HV). This is primarily attributed to the residual porosity (~4%) in the present pressureless-sintered samples, as pores act as stress concentrators and significantly degrade load-bearing capacity [29,30].

The mechanisms for hardness enhancement (≤15 wt.% CeO_2_) contain second-phase dispersion strengthening and grain refinement. Firstly, moderate CeO_2_ doping effectively inhibits grain growth (as evidenced in Figure 2), leading to grain refinement. This increases the density of grain boundaries, which act as barriers to dislocation motion [29,30]. Secondly, the dissolved Ce^4+^ ions form a supersaturated solid solution within the ZrO_2_ lattice. The associated lattice strain fields impede dislocation glide, enhancing hardness [27]. Thirdly, the finely dispersed CeO_2_ particles exert a pinning effect on grain boundaries, further stabilizing the refined microstructure and contributing to strengthening [27]. 

The mechanisms for hardness reduction (>15 wt.% CeO_2_) contain phase separation and stress concentration and increased porosity. Firstly, the excessive CeO_2_ exceeds its solid solubility limit, leading to secondary phase formation (CeO_2_ rich precipitates). These phases induce stress concentrations at grain boundaries due to thermal expansion mismatch and/or elastic modulus differences, facilitating crack initiation [31]. Secondly, as shown in Figure 2 and Figure 4, the porosity significantly increases beyond 15 wt.% CeO_2_. These pores act as stress concentrators and preferential sites for crack propagation, thereby diminishing the material’s load-bearing capacity and measured hardness [32].

**Figure 7:**
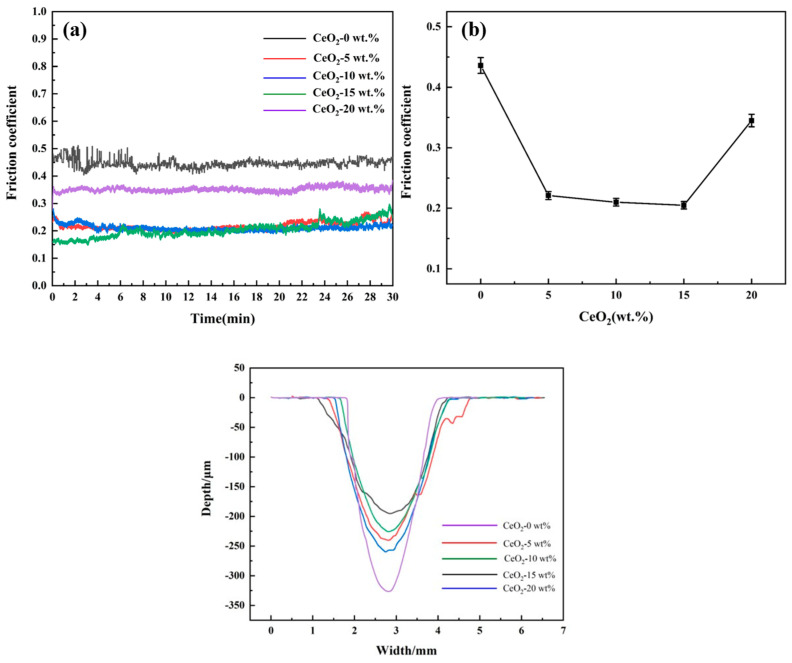
Friction coefficient curves (**a**), average friction coefficients (**b**), and 2D wear scar profiles of ZrO_2_ ceramic materials with different CeO_2_ doping contents.

Figure 7 presents the friction coefficient curves, average friction coefficients, and corresponding 2D wear scar profiles for ZrO_2_ ceramics doped with varying CeO_2_ contents. Complementary quantitative wear scar dimensions (width and depth) are provided in Table 2. As can be seen from Figure 7b, the average friction coefficient of CeO_2_-doped ZrO_2_ is the best when the CeO_2_ content is 15%, which is much lower than the optimal coefficient of 0.5–0.6 measured by Wen Deng et al. for 8YSZ [33]. For undoped ZrO_2_ (Figure 7a), it exhibits a prolonged friction coefficient stabilization period. This instability primarily arises from abrasive wear induced by surface micro-asperities and adhesive wear due to the absence of a self-lubricating phase [33], For CeO_2_-doped samples, it demonstrates significantly improved tribological performance. The 15Ce-ZrO_2_ sample achieves the lowest average friction coefficient (0.205, representing a 52.98% reduction compared to undoped ZrO_2_) and the shortest stabilization time (4 min). This enhancement is attributed to a synergistic effect. Firstly, CeO_2_ effectively seals intergranular defects and reduces surface roughness. Secondly, the enhanced hardness via solid solution strengthening and grain boundary pinning improves resistance to deformation and wear [20,25]. Thirdly, the inherent layered structure of CeO_2_ facilitates the development of a low-shear-strength lubricating transfer film at the sliding interface [20,25]. For excessive doping (>15 wt.% CeO_2_), the performance deteriorates due to two reasons. On the one hand, the agglomerated CeO_2_ particles detach, forming hard abrasive debris that accelerates wear. On the other hand, the reduced density and hardness diminish the wear resistance. Through wear scar analysis, the 15Ce-ZrO_2_ sample exhibits optimal wear resistance, with the smallest wear scar dimensions (width: 2.44 mm, depth: 71.17 μm, representing a 40.2% reduction in depth compared to undoped ZrO_2_). This is primarily a consequence of grain refinement and increased density, which reduces microcracking and the likelihood of third-body abrasive formation during sliding [34]. Conversely, the 20Ce-ZrO_2_ sample shows a significant rebound in wear depth (105.18 μm), directly correlating with its degraded density and hardness [33]. Optimal CeO_2_ doping (15 wt.%) synergistically enhances the tribological properties of ZrO_2_ ceramics through intergranular defect healing, microstructural strengthening, and effective tribo-film formation, minimizing friction and wear [33,35].

**Figure 8:**
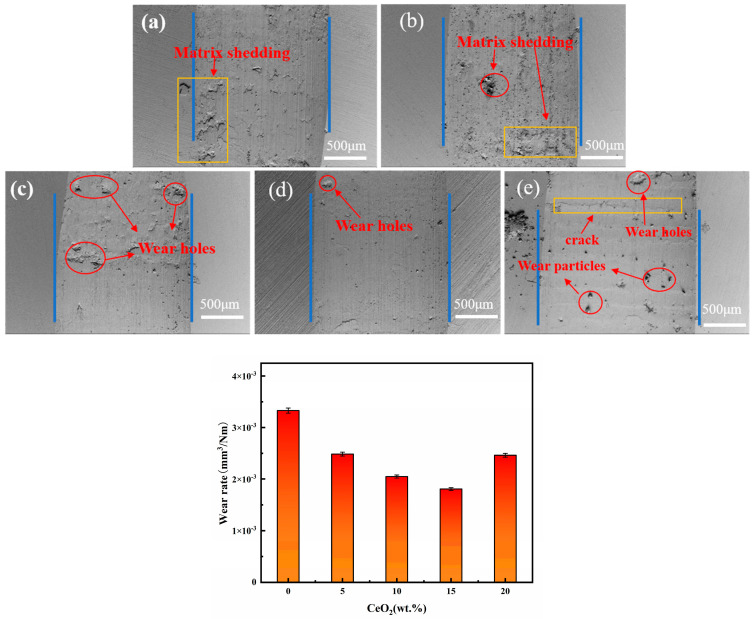
Wear rates and SEM images of wear scars for ZrO_2_ ceramics with different CeO_2_ doping contents: (**a**) 0Ce-ZrO_2_, (**b**) 5Ce-ZrO_2_, (**c**) 10Ce-ZrO_2_, (**d**) 15Ce-ZrO_2_, and (**e**) 20Ce-ZrO_2_.

**Figure 9:**
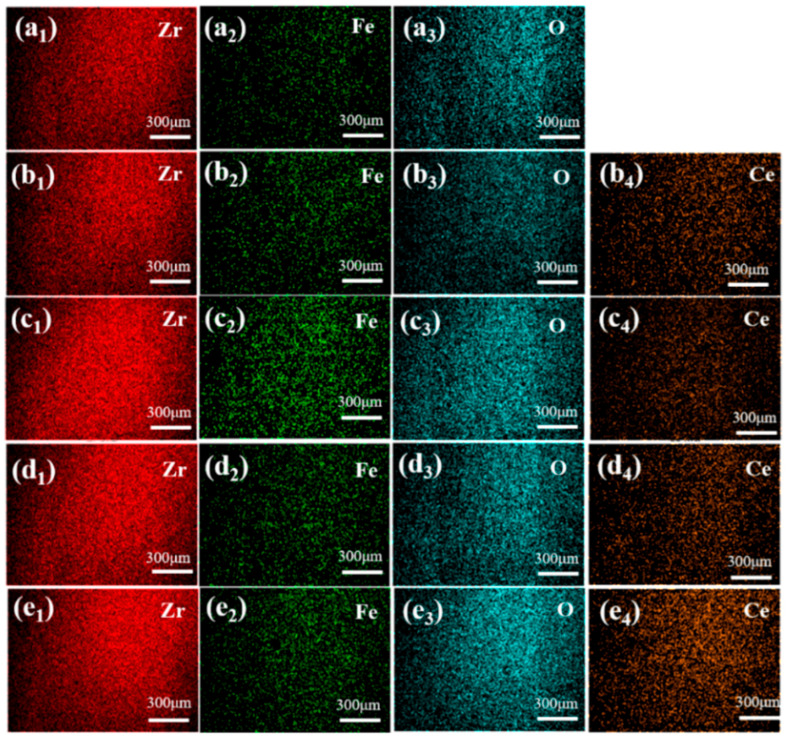
EDS elemental mapping of wear scars on CeO_2_-doped ZrO_2_ ceramics: (**a_1_**–**a_3_**) 0Ce-ZrO_2_, (**b_1_**–**b_4_**) 5Ce-ZrO_2_, (**c_1_**–**c_4_**) 10Ce-ZrO_2_, (**d_1_**–**d_4_**) 15Ce-ZrO_2_, and (**e_1_**–**e_4_**) 20Ce-ZrO_2_.

Figure 8 and Figure 9 present the SEM morphology of wear scars, corresponding EDS analysis, and wear rate data for ZrO_2_ ceramics with varying CeO_2_ doping contents. SEM wear scar analysis (Figure 8), All samples exhibit wear scars characterized by parallel grooves aligned with the sliding direction, accompanied by brittle delamination pits and debris accumulation. As CeO_2_ content increases from 0 to 15 wt.%, the severity of wear scars, microcracking density, and debris detachment progressively decrease. The 15Ce-ZrO_2_ sample demonstrates the smoothest wear surface. This is attributed to CeO_2_-induced grain refinement, microstructural homogenization, and defect density reduction [36]. Furthermore, the well-distributed CeO_2_ at grain boundaries effectively impedes dislocation motion and inhibits crack propagation, enhancing cohesive strength [20]. Conversely, the excessive doping (20 wt.% CeO_2_) promotes CeO_2_ agglomeration, increased porosity, and reduced hardness. These factors collectively lead to prominent transverse cracking and extensive abrasive debris generation within the wear scar [32]. From Figure 9, the wear rate decreases significantly from 3.33 × 10^−3^ mm^3^/(N·m) for undoped ZrO_2_ to 1.81 × 10^−3^ mm^3^/(N·m) for 15Ce-ZrO_2_, representing a 45.65% reduction. This enhancement correlates with solid solution strengthening and grain boundary pinning, which simultaneously elevate density and hardness. The 20Ce-ZrO_2_ sample exhibits a rebounded wear rate of 2.46 × 10^−3^ mm^3^/(N·m), primarily driven by grain boundary stress concentration (due to agglomeration/secondary phases) and elevated porosity [32].

**Figure 10:**
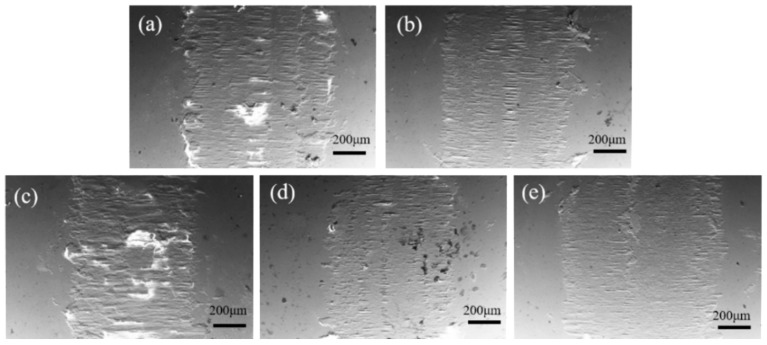
SEM images of wear scars on stainless steel balls’ counterfaces after sliding against (**a**) 0Ce-ZrO_2_, (**b**) 5Ce-ZrO_2_, (**c**) 10Ce-ZrO_2_, (**d**) 15Ce-ZrO_2_, and (**e**) 20Ce-ZrO_2_.

**Figure 11:**
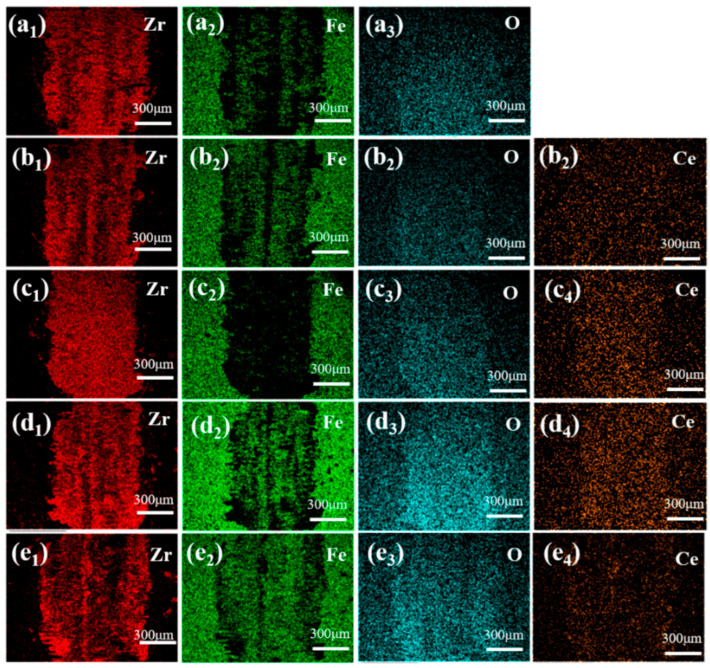
EDS diagram of wear marks on stainless steel ball surface: (**a_1_**–**a_3_**) 0Ce-ZrO_2_, (**b_1_**–**b_4_**) 5Ce-ZrO_2_, (**c_1_**–**c_4_**) 10Ce-ZrO_2_, (**d_1_**–**d_4_**) 15Ce-ZrO_2_, and (**e_1_**–**e_4_**) 20Ce-ZrO_2_.

Figure 10 and Figure 11 present the SEM morphology and corresponding EDS elemental mappings of worn surfaces following tribological testing. From wear scar morphology, all samples exhibit surfaces dominated by parallel grooves aligned with the sliding direction, accompanied by lamellar delamination debris accumulation. The 15Ce-ZrO_2_ sample demonstrates the narrowest/shallowest wear track and minimal debris accumulation. This correlates with grain boundary strengthening via CeO_2_ segregation/pinning to form a coherent, low-shear-strength lubricating tribo-film. Distinct Ce/Zr signals (co-localized with Fe/O) confirm adhesive material transfer from the counter-body under shear stress. However, excessive CeO_2_ (>15 wt.%) promotes hard phase agglomeration, which acts as stress concentrators. This, coupled with reduced hardness and increased porosity, exacerbates abrasive wear and surface fracture, leading to accelerated material removal.

### 3.4. Thermal Properties

**Figure 12:**
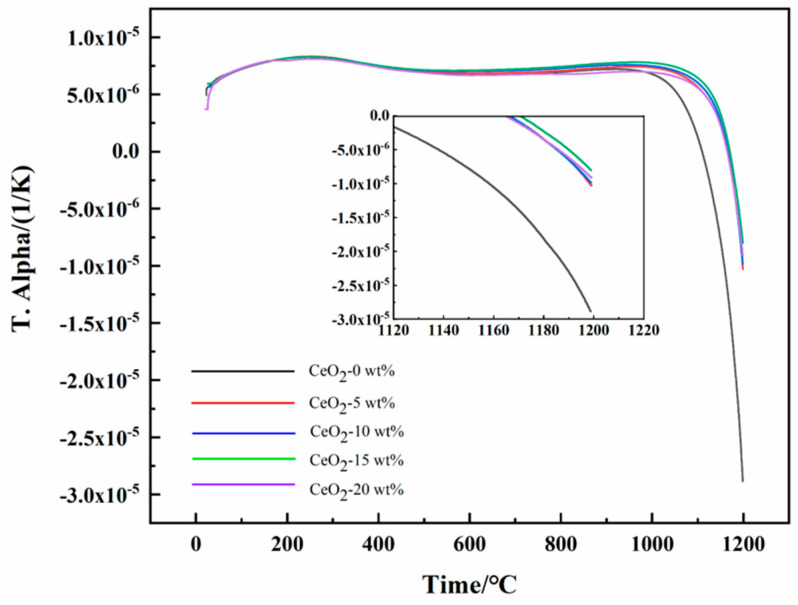
Coefficient of thermal expansion of ZrO_2_ ceramic materials doped with different contents of CeO_2_.

The coefficient of thermal expansion (CTE) is a critical performance parameter for zirconia ceramics, profoundly influencing their thermo-mechanical stability, manufacturability, and service performance in applications involving thermal cycling [37]. In Figure 12, both undoped ZrO_2_ and CeO_2_-doped ZrO_2_ exhibit rapid expansion below ~250 °C (CTE: ~8.818 × 10^−6^ K^−1^), followed by a gradual decline to 6.68–7.20 × 10^−6^ K^−1^ at 250–950 °C. The high congruence of CTE curves across all compositions in this range confirms that CeO_2_ doping preserves the intrinsic crystallographic stability of the ZrO_2_ matrix. CeO_2_ doping delays the onset of contraction to ≥1000 °C. Local magnification reveals drastically suppressed contraction magnitude in Ce-doped systems at 1120–1200 °C. Specifically, the 15Ce-ZrO_2_ achieves a 72.21% reduction in absolute CTE value at 1200 °C versus undoped ZrO_2_, demonstrating effective inhibition of high-temperature shrinkage. This significantly mitigates thermally induced mismatch stresses. The mechanism of shrinkage suppression was Ce^4+^ substitution (r = 0.97 Å, CN = 8) for Zr^4+^ (r = 0.84 Å, CN = 8) within the Zr_1−x_Ce_x_O_2_ solid solution. Firstly, the dilatational lattice strain from the larger Ce^4+^ ion expands the unit cell and reconstructs local stress fields. Secondly, charge compensation for Ce^4+^ incorporation is widely reported to promote the formation of oxygen vacancies in similar oxide systems. These vacancies are preferentially ordered along the c-axis, increasing the activation energy barrier for the reconstructive [38].

**Figure 13:**
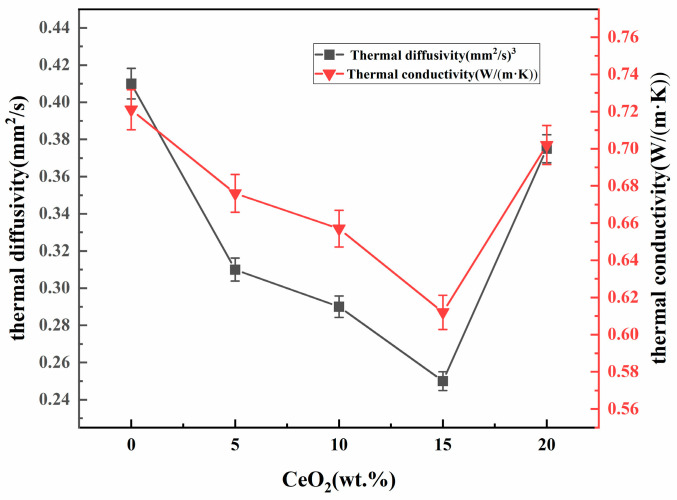
Thermal conductivity and thermal diffusivity of different CeO_2_-doped ZrO_2_ ceramics at 1100 °C.

The relative density-normalized thermal conductivity of 0–20 wt.% CeO_2_-doped samples is presented in Table 3. The thermal conductivity of ceramics is predominantly governed by phonon-mediated heat transfer [39,40,41,42]. Point defects which are commonly introduced by aliovalent doping, constitute potent sources of phonon scattering, arising from the associated mass defect and the disruption of interatomic bonding [43]. In ZrO_2_, its unique low thermal conductivity is attributed to lattice distortion and possible enhanced phonon scattering due to oxygen vacancies. These mechanisms reduce the thermal diffusivity, making zirconia an effective thermal barrier material [37]. Figure 13 shows the thermal conductivity of zirconium oxide doped with cerium oxide at a temperature of 1100 °C. At a cerium oxide content of 15% by mass, the lowest value (0.612 watts/(meter·kelvin)) is observed. This initial decrease may be attributed to the enhanced intrinsic phonon scattering caused by lattice strain and point defects associated with the doping of cerium^4+^ [44]. The subsequent increase in thermal conductivity coincides with a sharp rise in porosity beyond 15 wt.% (Figure 4). While pores scatter phonons, their detrimental impact on the heat conduction pathway can lead to complex effects on the net measured conductivity [45]. Combined with the potential stabilization of higher thermal conductivity phases, the degraded, porous microstructure at 20 wt.% CeO_2_ explains the observed rebound.

## 4. Conclusions

Building upon the established optimal doping range (~10–15 wt.% CeO_2_), this work systematically investigates the composition-dependent evolution of microstructure and mechanical, tribological, and thermal properties in ZrO_2_ ceramics across 0–20 wt.% CeO_2_. The 15 wt.% CeO_2_ composition achieves the best performance balance: hardness increases by 27.6% to 310 HV_1_, friction coefficient decreases to 0.205, wear rate drops to 1.81 × 10^−3^ mm^3^/(N·m), thermal expansion at 1200 °C is reduced by 72.2%, and thermal conductivity reaches 0.612 W/(m·K). These improvements are primarily attributed to solid solution strengthening and grain refinement, while the lowered thermal conductivity is consistent with enhanced phonon scattering, potentially involving point defects such as oxygen vacancies. Beyond 15 wt.%, performance declines due to CeO_2_ agglomeration, duplex structure formation, and increased porosity. This study thus validates the optimal doping level through systematic evaluation and provides a composition structure property framework to guide the design of multifunctional ZrO_2_ ceramics.

## Figures and Tables

**Table 1:** Experimental formulations.

No.	Sample Name	ZrO_2_ (wt.%)	CeO_2_ (wt.%)
1	ZrO_2_	100	0
2	5Ce-ZrO_2_	95	5
3	10Ce-ZrO_2_	90	10
4	15Ce-ZrO_2_	85	15
5	20Ce-ZrO_2_	80	20

**Table 2:** Width and depth of wear scars for ZrO_2_ ceramic materials with different CeO_2_ doping contents.

CeO_2_ Doping Amount (wt.%)	Width (mm)	Depth (μm)
0	2.21	132.15
5	2.86	97.26
10	2.65	91.36
15	2.44	71.17
20	2.72	105.18

**Table 3:** Thermal conductivity of 0–20 wt.% CeO_2_-doped zirconia after relative density normalization.

CeO_2_ (wt.%)	0	5	10	15	20
relative density (%)	94.4	95.1	95.4	96.1	94.8
thermal conductivity (W/(m·K))	0.726	0.675	0.658	0.612	0.702
revised thermal conductivity (W/(m·K))	0.685	0.642	0.627	0.588	0.665

## Data Availability

The original contributions presented in this study are included in the article. Further inquiries can be directed to the corresponding authors.
